# The Prevalence of *Staphylococcus aureus* and the Occurrence of MRSA CC398 in Monkey Feces in a Zoo Park in Eastern China

**DOI:** 10.3390/ani11030732

**Published:** 2021-03-08

**Authors:** Yuanyue Tang, Zhuang Qiao, Zhenyu Wang, Yang Li, Jingwei Ren, Liang Wen, Xun Xu, Jun Yang, Chenyi Yu, Chuang Meng, Hanne Ingmer, Qiuchun Li, Xinan Jiao

**Affiliations:** 1 Key Laboratory of Prevention and Control of Biological Hazard Factors (Animal Origin) for Agri-Food Safety and Quality, Ministry of Agriculture of China, Yangzhou University, Wenhui East Road 48, Yangzhou 225009, China; tangyy@yzu.edu.cn (Y.T.); MX120180689@yzu.edu.cn (Z.Q.); DX120200156@yzu.edu.cn (Z.W.); mx120170799@yzu.edu.cn (Y.L.); 18752731442@163.com (J.R.); mengchuang@yzu.edu.cn (C.M.); 2 Jiangsu Key Lab of Zoonosis/Jiangsu Co-Innovation Center for Prevention and Control of Important Animal Infectious Diseases and Zoonoses, Yangzhou University, Wenhui East Road 48, Yangzhou 225009, China; 3 Joint International Research Laboratory of Agriculture and Agri-Product Safety, Yangzhou University, Wenhui East Road 48, Yangzhou 225009, China; 4 Yangzhou Ecological Zoo, Zhu Yu Wan Road 888, Yangzhou 225009, China; wenliang202102@163.com (L.W.); yzboyxx@163.com (X.X.); yangjun20210221@126.com (J.Y.); chenyiyu2021@126.com (C.Y.); 5 Department of Veterinary and Animal Science, Faculty of Health and Medical Sciences, University of Copenhagen, 1870 Frederiksberg, Denmark; hi@sund.ku.dk

**Keywords:** non-human primates, *Staphylococcus aureus*, MRSA, CC398

## Abstract

**Simple Summary:**

The increasing observation of methicillin-resistant *Staphylococcus aureus* (MRSA) in wildlife species has raised the concern of its impact on the animal health and the potential of zoonotic transmission. The molecular types of MRSA and *S. aureus* in wild animals has been found to mix with several molecular types from humans and livestock, indicating a dynamics of *S. aureus* transmission between these animals and humans. Thus, it is important to investigate the epidemiological relationship of MRSA between zoo animals and humans. To investigate the prevalence and transmission of *S. aureus* in non-human primates and their living environment, we conducted this study on all the non-human primates in a zoo park located in eastern China. Fecal samples from non-human primates and samples from their living environments were selectively enriched and conducted for *S. aureus* isolation. The whole genome analysis showed that these two MRSA t034/CC398 isolates were found from a monkey fecal sample and its living environment. The virulence factor analysis showed that these two MRSA isolates carried virulence factors relating to the cell adherence, biofilm formation, toxins, and human-associated immune evasion cluster. The further phylogenetic analysis revealed that these two MRSA isolates have a close genomic relationship with the human-associated clinical isolates from China, which indicates a potential risk of bidirectional transfer of MRSA between monkeys and humans.

**Abstract:**

Methicillin-resistant *Staphylococcus aureus* (MRSA) is one of the important antibiotic resistant pathogens causing infections in humans and animals. The increasing observation of MRSA in wildlife species has raised the concern of its impact on animal health and the potential of zoonotic transmission. This study investigated the prevalence of *S. aureus* in fecal samples from non-human primates in a zoo located in Jiangsu, China, in which 6 out of 31 (19.4%) fecal samples, and 2 out of 14 (14.3%) indoor room floor swab samples were *S. aureus*-positive. The antibiotic susceptibility tests of the eight isolates showed that the two isolates were resistant to both penicillin and cefoxitin, the three isolates were resistant only to penicillin, while three isolates were susceptible to all detected antibiotics. The two isolates resistant to cefoxitin were further identified as MRSA by the presence of *mecA*. Five different *spa* types were identified including t034 of two MRSA isolates from *Trachypithecus francoisi*, t189 of two methicillin-susceptible *S. aureus* (MSSA) isolates from *Rhinopithecus roxellana*, t377 of two MSSA isolates from *Colobus guereza*, and two novel *spa* types t19488 and t19499 from *Papio anubis*. Whole genome sequencing analysis showed that MRSA t034 isolates belonged to ST398 clustered in clonal complex 398 (CC398) and carried the type B ΦSa3 prophage. The phylogenetic analysis showed that the two MRSA t034/ST398 isolates were closely related to the human-associated MSSA in China. Moreover, two MRSA isolates contained the virulence genes relating to the cell adherence, biofilm formation, toxins, and the human-associated immune evasion cluster, which indicated the potential of bidirectional transfer of MRSA between monkeys and humans. This study is the first to report MRSA CC398 from monkey feces in China, indicating that MRSA CC398 could colonize in monkey and have the risk of transmission between humans and monkeys.

## 1. Introduction

Methicillin-resistant *Staphylococcus aureus* (MRSA) is an important antimicrobial-resistant pathogen that can cause acute and chronic infections in animals and humans [1,2]. *S. aureus* obtains the resistance to β-lactams by an alternative penicillin-binding protein encoded by the *mec* gene. Human-associated MRSA has been epidemiologically divided into the hospital-associated MRSA (HA-MRSA) and community-associated MRSA (CA-MRSA), while a distinct group of livestock-associated MRSA (LA-MRSA) has been associated with animals. Increasing public health concern has been raised due to the transmission of LA-MRSA from animals to humans via direct or indirect contact with animals, animal origin products, or animal-related environment materials [3,4]. LA-MRSA clonal complex 398 (CC398) has taken a special concern, one that is widely detected in pigs and has been frequently reported causing human infections through close contact with contaminated livestock or food products [5,6,7].

*S. aureus* CC398 has been epidemiologically divided into two subpopulations, which are the livestock-associated clade carrying SCC*mec* and *tet(M)* and the human-associated clade containing the β-hemolysin negatively converting prophage ΦSa3 with the human-specific immune evasion cluster (IEC) [8]. In addition, *spa* typing targeting to the polymorphic X region of the *spa* gene is also a commonly used subtyping method to provide the genetic basis for the epidemiological study [9]. In China, methicillin-susceptible *S. aureus* (MSSA) CC398 with *spa* type t011, t899, and t034 have been more frequently reported to be the cause of human infections, occupying approximately 20% of the infection cases in the hospital [10]. MRSA CC398 has also been reported to infect children and patients in Chinese hospitals [11,12]. *S. aureus* CC398 has been reported as one of the predominate CC types causing bovine mastitis [13]. In China, the predominate *S. aureus* belongs to CC9 *spa* type t899 in pig, while *S. aureus* CC398 has only been sporadically detected in pig and retail food samples [9]. 

Besides being a pathogen in humans and livestock, *S. aureus* also colonizes companion animals and wildlife species and could act as an opportunistic pathogen in many of these hosts [14]. The molecular types of MRSA and MSSA in wild animals have been found to mix with several molecular types from humans and livestock, indicating a dynamics of *S. aureus* transmission between different species, which could be crucial risk factors for new pandemics [15]. MRSA and MSSA in non-human primates have been sporadically reported in the USA, Africa, and China [16,17,18]. MRSA ST188 and ST3268 have been known as the predominant ST types from macaques in both the USA and China, which have been detected both in nares and intestinal tracts [15,18]. 

Nares have been traditionally regarded as the main niche of *S. aureus* colonization. However, *S. aureus* colonizing in intestinal tracts has also raised concerns, especially in human infants and children [19]. *S. aureus* has been reported to colonize the intestinal tract with an average frequency of approximately 20%, while 9% is identified as MRSA, which occupied nearly half of the nasal carriage [20]. The detection of *S. aureus* or MRSA in intestinal tracts has also been reported in chimpanzees, bats, rats, and red deer, indicating that animal intestinal tracts could be the natural habitat for *S. aureus* [15]. Of greater concern, the detection of *S. aureus* in fecal samples from slaughtered reindeer in Finland and Norway indicate that animals involved in human life could be the potential carriers for *S. aureus* transmission in humans [15].

This study investigated the prevalence of *S. aureus* and MRSA in non-human primates from a zoo in Jiangsu, China. The surveillance study for fecal samples from animals and environmental samples provided the information for the distribution of *S. aureus* in the animal activity area in the zoo park. The whole genome sequence analysis was conducted to reveal the molecular types and virulent characteristics of MRSA isolates, while the phylogenetic analysis further revealed the genetic relationship of MRSA isolates to human-associated *S. aureus*. 

## 2. Materials and Methods

### 2.1. Sample Collection and Bacterial Isolation

Samples from non-human primate animals were collected in January 2019 at Yangzhou Ecological Zoo in Jiangsu, China. A total of 55 samples were collected, including 31 fecal samples, 10 environmental swab samples, and 14 food samples from their indoor living environment (Table S1). All samples were collected early in the morning from the animal indoor room area after animals were released to outdoor activity areas. All fecal samples were immediately stored in fecal collection tube (SC-2, Bang Shuo, Guangzhou, China). All environment swab samples were collected by cotton swab immersed with phosphate-buffered saline (PBS) and stored in the sterilized sample bags. Food samples were collected by one-time sterilized gloves and stored in sterilized sample bags. All samples were subjected to bacterial isolation within 24 h in the laboratory. The study was conducted according to the Guide for the Care and Use of Laboratory Animals of the Ministry of Health (SYXK[Su] 2017-0045), China, with the permission from the Research Ethics Committee of Yangzhou University, Jiangsu, China.

*S. aureus* and MRSA were isolated as previously described [18]. Briefly, all fecal samples were inoculated in 5 mL trypticase soy broth (TSB; Qingdao Hope Bio-Technology Co., Ltd. Shangdong, China) with 6.5% NaCl, while all environment swab samples and food samples were full mixed with 15 mL TSB with 6.5% NaCl. All samples were enriched overnight at 37 °C, 180 rpm. Ten microliter aliquots of each enriched sample were inoculated on CHROMagar Staph aureus plates for *S. aureus* selection and CHROMagar MRSA plates for MRSA selection (CHROmagar, Paris, France). All agar plates were incubated at 37°C overnight, and presumptive *S. aureus* clones were confirmed by PCR analysis for the presence of *nuc* and *mecA* as previously described [21].

### 2.2. Antimicrobial Susceptibility Test

Antimicrobial susceptibility testing was performed by the disc diffusion method according to the standard of Clinical and Laboratory Standards Institute (CLSI) VET01 [22]. Each isolate was tested with 13 antimicrobial agents, namely, cefoxitin (FOX), erythromycin (E), tetracycline (TE), chloramphenicol (C), ciprofloxacin (CIP), clindamycin (DA), gentamicin (CN), kanamycin (K), linezolid (LZD), nitrofurantoin (F), penicillin (P), rifampin (RD), and trimethoprim (SXT). *S. aureus* ATCC29213 was included as the quality control. Each experiment was repeated in triplicate.

### 2.3. spa Typing

*S. aureus* isolates from each sample was analyzed for the *spa* type. PCR program for the *spa* gene was performed with primers *spa*-1113f and *spa*-1514f [23], and PCR products were sequenced by Genscript Biotech Corporation, (Nanjing, China). The *spa* type for each isolate was analyzed by Ridom Spa Server database (http://spaserver.ridom.de/, accessed on 14 November 2020). 

### 2.4. Whole Genome Sequencing of MRSA Isolates and Comparative Genomic Analysis

Positive *S. aureus* colonies on CHROMagar MRSA plates from each sample were analyzed for *mecA* and *mecC* by PCR program [24]. The identified MRSA isolates were whole genome-sequenced using the Hiseq2500 platform (Illumina, San Diego, California, USA) with 500 cycles of paired reads. Raw reads were trimmed and filtered by NGSQC toolkit and assembled by SPAdes 3.6 with de novo assembly and the subsequent annotation of the assembled sequences by Prokka version 1.12. Whole genome sequence (WGS) data of 2 MRSA isolates were submitted to the European Nucleotide Archive database with the accession number PRJEB42195. The multi-locus sequence types were obtained by submitting the WGS data to *S. aureus* multi-locus sequence typing (MLST) database (https://pubmlst.org/saureus/, accessed on 14 November 2020). Scc*mec* types of both MRSA isolates were analyzed by SCCmecFinder 1.2 (https://cge.cbs.dtu.dk/services/SCCmecFinder-1.2/, accessed on 14 November 2020). Core genome MLST (cgMLST) was conducted for MRSA isolates and the previously reported 28 *S. aureus* t034/CC398 isolates (Table S3) [25], and the phylogenetic tree were constructed to reveal the genetical relationship of all *S. aureus* t034/CC398 strains. To evaluate the virulent characteristics of MRSA isolates, we identified virulence factors relating to the cell adherence, biofilm formation, and toxins by basic local alignment search tool for nucleotides (blastn) including *clfA* and *clfB* for clumping factors; *cna* for collagen-binding protein; *ebp* for elastin-binding protein; *fnbA* and *fnbB* for fibronectin binding proteins; *icaA*, *icaB*, *icaC*, *icaD*, and *icaR* for intercellular adhesion proteins; *sdrC* for Ser-Asp rich protein; *hly/hla* for α-hemolysin; *hlb* for β-hemolysin; *hld* for δ-hemolysin; *lukF-PV* and *lukS-PV* for Panton-Valentine leucocidin (PVL); and *chp*, *scn*, and *sak* for the immune evasion cluster.

## 3. Results and Discussion

Out of 31 fecal samples, 6 (19.4%) from non-human primates were *S. aureus* isolates, including two isolates from *Colobus guereza*, two isolates from four *Papio Anubis*, one isolate from four *Trachypithecus francoisi*, and one isolate from *Rhinopithecus roxellana*, and no *S. aureus* was observed from *Hylobates lar* (Tables S1 and S2). Among 24 environmental samples, 2 out of 14 (14.3%) *S. aureus* isolates were detected from the indoor room floor swab samples of *Rhinopithecus roxellana* and *Trachypithecus francoisi*. No contamination of *S. aureus* was observed from animal food samples. Among the eight *S. aureus* isolates, two isolates were identified as MRSA, one from *Trachypithecus francoisi* fecal samples and one from the indoor room floor swab sample of *Trachypithecus francoisi*. Our previous study showed that the prevalence of *S. aureus* was 26% and MRSA was 5% from macaque fecal samples [18], which is higher than 13% (6/45) of *S. aureus* and 2% (1/45) of MRSA from six different species of monkeys in this study. The prevalence of *S. aureus* is close to the reported prevalence of 19% in non-human primates from African parks, while the prevalence of MRSA was 5.3% and 1.7% in Cote d’Ivoire and DR Congo regions, respectively [26]. This study also detected MRSA and MSSA from the indoor room floor swab samples, which is correspondent to the previous study that MRSA could contaminate the primate environmental facility samples [27]. 

The antimicrobial susceptibility test revealed that the two MRSA isolates were resistant to both penicillin and cefoxitin, while the three MSSA isolates were resistant to penicillin, and the other three MSSA isolates were susceptible to all detected antibiotics (Table 1). Five *spa* types were identified from the eight *S. aureus* isolates, including two MRSA t034 isolates from *Trachypithecus francoisi* and two MSSA t189 isolates from *Rhinopithecus roxellana*, the fecal samples and the indoor room floor from both, and two MSSA t377 isolates from fecal samples of *Colobus guereza*. Moreover, two novel *spa* types t19488 and t19499 were identified in two MSSA isolates from two *Papio anubis* fecal samples. The *spa* type t189 was reported as one of the predominant genotypes of *S. aureus* from a non-human primate, which was detected in macaque, chimpanzee, and lemur [16,18,26,28], while *spa* type t377 has not been reported in wildlife species until now [15]. MSSA t377 was reported as a predominant *S. aureus* strain in hospitals, sporadically causing human clinical infections in China, which was also confirmed with a relatively high biofilm formation ability for its persistence and transmission in the environment [29]. Notably, MLST analysis showed that both of two MRSA t034 isolates belonged to ST398, which was not reported in any non-human primates before and was only detected from Norway rats and wild boars [15]. In addition, both MRSA isolates belonged SCC*mec* V (5C2&5), which were commonly observed as relating to MRSA ST398/t034 [30].

**Figure 1:**
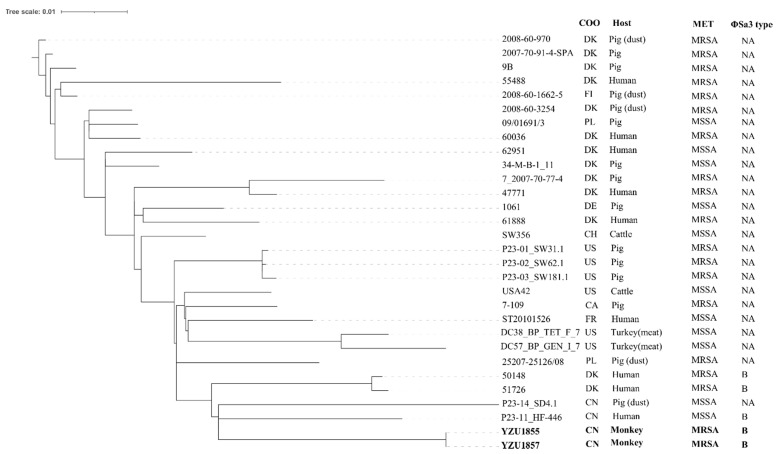
The phylogenetic tree of the *Staphylococcus aureus* t034/CC398 in terms of the core genome multi-locus sequence type (cgMLST). Strain YZU1855 and YZU1857 (bold font) were MRSA t034/CC398 from this study, while other strains were from a previous study (Table S3) [25]. COO, the country of origin; CA, Canada; CH, Switzerland; CN, China; DE, Germany; DK, Denmark; FI, Finland; FR, France; PL, Poland; US, United States.

The genome sequence analysis showed that both MRSA t034/ST398 isolates YZU1855 and YZU1857 contained the *mecA* gene and the type B ΦSa3 prophage carrying *sak*, *chp*, and *scn*. We further investigated the phylogenetic relationship of two MRSA t034/ST398 isolates to human-associated MRSA and LA-MRSA by comparing to 28 *S. aureus* t034/ST398 isolates from a previously published study [25] (Figure 1; Table S3). According to the core genome analysis, both YZU1855 and YZU1857 from this study were closely related to the MSSA isolate P23-10_WZ-103 from humans in China (Figure 1). The two MRSA isolates showed the same virotype relating to the cell adherence, toxin, and the human immune invasion cluster, including *clfB* for clumping factor [31]; *ebp* for elastin-binding protein [32]; *fnbA* for fibronectin-binding proteins [33]; *icaA*, *icaB*, *icaC*, *icaD*, and *icaR* for intercellular adhesion proteins and biofilm formation [34]; *hla* for α-hemolysin [35]; *chp* for chemotaxis inhibitory protein [36]; *scn* for staphylococcal complement inhibitor [37]; and *sak* for staphylokinase [38] (Table 1). The presence of the cell adherence factors and the human immune invasion clusters indicated that the two MRSA t034/CC398 had the potential of bi-direction transfer between monkeys and animals. *lukF-PV* and *lukS-P* for Panton–Valentine leucocidin were not detected in MRSA isolates. The presence of PVL has been commonly associated with isolates from community and health care. However, a previous study showed that only 17.1% of MRSA ST398 from China harboured *lukS/F-PV* [39]. The occurrence of human-associated MRSA t034/ST398 in monkey feces provided evidence of MRSA CC398 colonizing in the monkey gastrointestinal tract. Moreover, our study also showed the occurrence of MRSA and MSSA from both monkey fecal samples and its indoor living environment, indicating that the direct contact to animal and the contaminated environment could increase the risk of *S. aureus* transmission frequency [27,40].

## 4. Conclusions

This study is the first report on MRSA CC398 recovered from monkeys in China. The identification of two MRSA t034/ST398 isolates in monkey feces suggests that MRSA CC398 can colonize the monkey gastrointestinal tract. The phylogenetic analysis demonstrated a close relationship of MRSA t034/ST398 to the human-associated CC398, suggesting the potential of MRSA CC398 to be transmitted between humans and animals. Further investigation on the genetic relationship of *S. aureus* isolates from monkeys and humans would be important to understand the potential of bidirectional transmission of *S. aureus*.

## Figures and Tables

**Table 1:** The antimicrobial resistant profile and genomic characterization of *Staphylococcus* *aureus* isolates.

Isolate No.	Animal Species	Origin	*spa* Type	SCC*mec* Type	Antibiotic Resistant Profile	MRSA/MSSA	Virulotype
LQSA19337	*Colobus guereza*	feces	t377	-	P	MSSA	ND ^a^
LQSA19342	*Colobus guereza*	feces	t377	-	P	MSSA	ND
LQSA19339	*Rhinopithecus roxellana*	floor	t189	-	-	MSSA	ND
LQSA19343	*Rhinopithecus roxellana*	feces	t189	-	P	MSSA	ND
LQSA19340	*Papio anubis*	feces	t19448	-	-	MSSA	ND
LQSA19341	*Papio anubis*	feces	t19449	-	-	MSSA	ND
YZU1855	*Trachypithecus francoisi*	floor	t034	V (5C2&5)	P-FOX	MRSA	*icaA*, *icaB*, *icaC*, *icaD*, *icaR*, *clfB*, *ebp*, *fnbA*, *sdrC*, *chp*, *scn*, *sak*
YZU1857	*Trachypithecus francoisi*	feces	t034	V (5C2&5)	P-FOX	MRSA	*icaA*, *icaB*, *icaC*, *icaD*, *icaR*, *clfB*, *ebp*, *fnbA*, *sdrC*, *chp*, *scn*, *sak*

^a^ ND: not detected.

## Data Availability

The datasets for this manuscript are not publicly available because the results of this manuscript have not been published yet. Requests to access the datasets should be directed to Yuanyue Tang.

## Supplementary Materials

The following are available online at https://www.mdpi.com/2076-2615/11/3/732/s1: Table S1: Sampling information of monkey feces and prevalence of *S. aureus*. Table S2: Sampling information of environment and prevalence of *S. aureus*. Table S3: Isolates information of *S. aureus* from the study by Price et al. [25].
